# The Large Zinc Finger Protein ZAS3 Is a Critical Modulator of Osteoclastogenesis

**DOI:** 10.1371/journal.pone.0017161

**Published:** 2011-03-03

**Authors:** Shujun Liu, Francesca Madiai, Kevin V. Hackshaw, Carl E. Allen, Joseph Carl, Emily Huschart, Chris Karanfilov, Alan Litsky, Christopher J. Hickey, Guido Marcucci, Sarandeep Huja, Sudha Agarwal, Jianhua Yu, Michael A. Caligiuri, Lai-Chu Wu

**Affiliations:** 1 Department of Internal Medicine, The Ohio State University, Columbus, Ohio, United States of America; 2 Comprehensive Cancer Center, The Ohio State University, Columbus, Ohio, United States of America; 3 Molecular and Cellular Developmental Biology Graduate Program, The Ohio State University, Columbus, Ohio, United States of America; 4 Department of Molecular and Cellular Biochemistry, The Ohio State University, Columbus, Ohio, United States of America; 5 Integrated Biomedical Science Graduate Program, The Ohio State University, Columbus, Ohio, United States of America; 6 Department of Orthopaedics and Department of Biomedical Engineering, The Ohio State University, Columbus, Ohio, United States of America; 7 Division of Orthodontics, The Ohio State University, Columbus, Ohio, United States of America; 8 Biomechanics and Tissue Engineering Laboratory, Division of Oral Biology, The Ohio State University, Columbus, Ohio, United States of America; Instituto Nacional de Câncer, Brazil

## Abstract

**Background:**

Mice deficient in the large zinc finger protein, ZAS3, show postnatal increase in bone mass suggesting that ZAS3 is critical in the regulation of bone homeostasis. Although ZAS3 has been shown to inhibit osteoblast differentiation, its role on osteoclastogenesis has not been determined. In this report we demonstrated the role of ZAS3 in bone resorption by examining the signaling mechanisms involved in osteoclastogenesis.

**Methodology/Principal Findings:**

Comparison of adult wild-type and ZAS3 knockout (ZAS3−/−) mice showed that ZAS3 deficiency led to thicker bones that are more resistant to mechanical fracture. Additionally, ZAS3−/− bones showed fewer osteoclasts and inefficient M-CSF/sRANKL-mediated osteoclastogenesis ex vivo. Utilizing RAW 264.7 pre-osteoclasts, we demonstrated that overexpression of ZAS3 promoted osteoclastogenesis and the expression of crucial osteoclastic molecules, including phospho-p38, c-Jun, NFATc1, TRAP and CTSK. Contrarily, ZAS3 silencing by siRNA inhibited osteoclastogenesis. Co-immunoprecipitation experiments demonstrated that ZAS3 associated with TRAF6, the major receptor associated molecule in RANK signaling. Furthermore, EMSA suggested that nuclear ZAS3 could regulate transcription by binding to gene regulatory elements.

**Conclusion/Significance:**

Collectively, the data suggested a novel role of ZAS3 as a positive regulator of osteoclast differentiation. ZAS3 deficiency caused increased bone mass, at least in part due to decreased osteoclast formation and bone resorption. These functions of ZAS3 were mediated via activation of multiple intracellular targets. In the cytoplasmic compartment, ZAS3 associated with TRAF6 to control NF-kB and MAP kinase signaling cascades. Nuclear ZAS3 acted as a transcriptional regulator for osteoclast-associated genes. Additionally, ZAS3 activated NFATc1 required for the integration of RANK signaling in the terminal differentiation of osteoclasts. Thus, ZAS3 was a crucial molecule in osteoclast differentiation, which might potentially serve as a target in the design of therapeutic interventions for the treatment of bone diseases related to increased osteoclast activity such as postmenopausal osteoporosis, Paget's disease, and rheumatoid arthritis.

## Introduction

The large zinc finger protein, ZAS3, is a member of the *ZAS/HIVEP/schnurri (shn)* protein family, which encodes unusually large transcriptional proteins of more than 250 kDa in size [reviewed in 1], [2]. ZAS3 has been cloned by screening expression cDNA libraries using the V(D)J recombination signal sequences or the calcium-binding protein *S100A4/mts-1* gene enhancer [3], [4]. The ZAS proteins are named after a composite protein structure, the ZAS domain, which consists of a pair of consecutive C_2_H_2_ **z**inc fingers, an **a**cidic domain and a **s**er/thr-rich region [2]. Zinc fingers and acidic domains are known structures for DNA binding and protein-protein interaction, respectively. Each ZAS protein contains two widely separated ZAS domains. Individual ZAS domains have been shown to bind specifically to *cis*-acting gene regulatory elements with a consensus sequence GGGGNNNNCC [2].

Cumulating evidence suggests that ZAS proteins play diverse cellular functions. Initial studies focusing on the DNA binding and transcriptional activities of the ZAS proteins have identified several ZAS target genes involved in growth, immunity, and development, including interferon-β [5], collagen type II [6], somatostatin type II receptor [7], c-myc [8], and S100A4/mts1 [4]. In addition, ZAS proteins associate with adaptor molecules to regulate signal transduction of the Smad complex in the bone morphogenetic protein (BMP) and transforming growth factor (TGF)-β pathways, and TRAF2 in the TNFα signaling cascades [9]–[12]. ZAS3 can also associate with the transcription factor c-Jun to augment AP-1-mediated *IL-2* expression [13]. Recently, ZAS proteins have also been shown to affect protein stability. ZAS2 (Shn-2) associates with chloride intracellular channel 4 to stabilize phospho-Smad2/3, whereas ZAS3 associates with the E3 ubiquitin ligase WWP1 to facilitate the degradation of Runx2, the principal transcriptional regulator of osteoblast differentiation [10], [14]. The diverse functions of the ZAS proteins in the regulation of transcription, signal transduction and protein turnover suggest that they likely play important roles in many physiological processes.

To investigate the physiological functions of the ZAS proteins, single and double knockout mice for *ZAS2* and *ZAS3* have been generated, which reveal that both genes are important for postnatal bone development and endochondral ossification [14]–[17]. With respect to bone homeostasis, the lack of ZAS2 suppresses both osteoclast and osteoblast activities, and generally, the suppression of osteoblast activities overrides that of osteoclasts as there is an overall reduction of bone mass in *ZAS2*−/− mice [16]. On the other hand, adult *ZAS3−/−* mice have a higher bone mass due to augmented osteoblast activity [14], [15].

Skeletal homeostasis, including adult bone mass, is determined by the balanced activities of two specific cell types; bone forming osteoblasts and bone resorbing osteoclasts. Therefore, the possibility that the increased bone mass in *ZAS3−/−* mice is due to osteoclast deficiency exists. We speculated that ZAS3 might regulate osteoclasts based on the following considerations: (i) ZAS3 is expressed in monocytes/macrophages, which share a common hematopoietic progenitor with osteoclasts [3]; (ii) ZAS3 can affect gene expression by association with c-Jun [13], an essential transcription factor for osteoclastogenesis [18], [19]; and (iii) The DNA binding activities of NF-kB and AP-1, important transcriptional mediators for osteoclastogenesis, exhibit tissue-specific alterations in *ZAS3−/−* mice [15], [20]. Therefore in this report, we have characterized osteoclasts in *ZAS3−/−* mice, and examined the role of ZAS3 in osteoclastogenesis. We showed that (i) bones of adult *ZAS3−/−* mice had fewer osteoclasts; (ii) ZAS3 was upregulated upon osteoclastogenesis of bone marrow macrophages (BMM) as well as of RAW264.7 pre-osteoclasts; (iii) sRANKL induced inefficient differentiation of *ZAS3−/−* BMM into osteoclast-like cells (OLCs) as compared to wild-type (WT) BMM; and (iv) forced expression of ZAS3 promoted, whereas silencing of ZAS3 inhibited, sRANKL-induced differentiation of RAW 264.7 preosteoclasts into osteoclasts and the expression of osteoclast-associated genes. Hence, the data identified a novel physiological function of ZAS3 in osteoclast biology and in adult bone remodeling.

## Results

### *ZAS3*−/− mice exhibit thicker bones with increased fracture resistance

**Figure 1:**
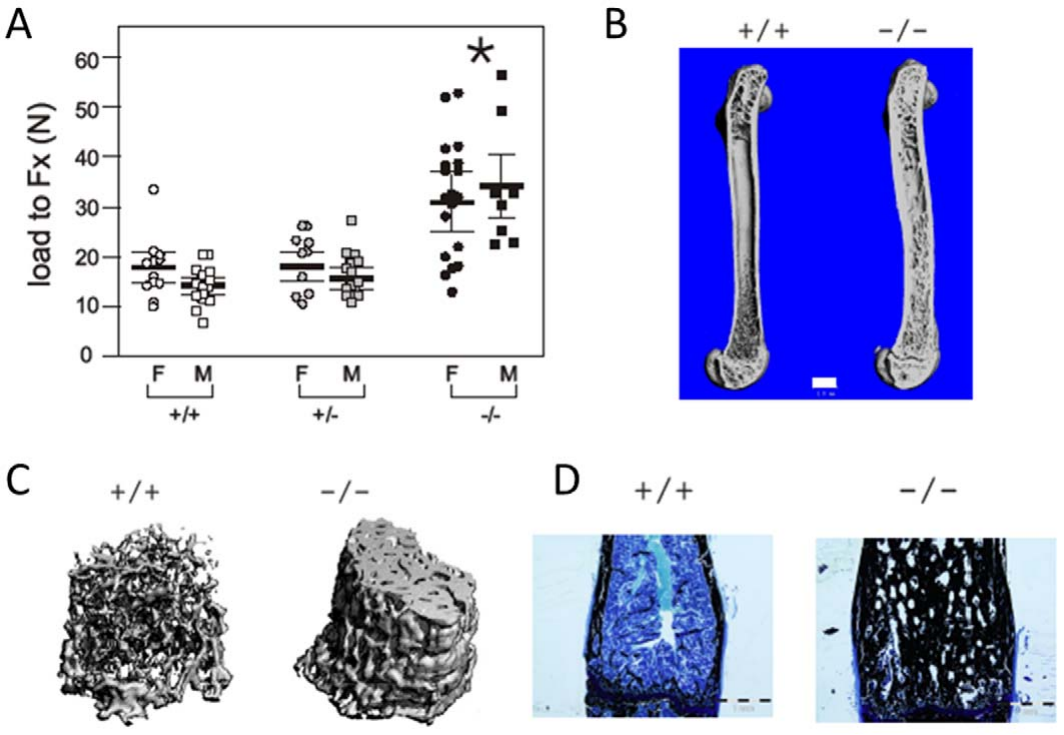
The bones of adult *ZAS3* knockout mice have increased bone strength, thickness, and mineralization. (A) Biomechanical properties of femurs were evaluated by three-point bending test. Load to fracture (Fx) was significantly increased in both male and female *ZAS3−/−* mice compared to +/+ and +/− control mice (*, *p*<0.05). (B) & (C) Micro-CT reconstruction images of femora of 4-month-old mice. Bar represents 1 mm. (D) Sagittal section of distal femora from 5-month-old mice stained with von Kossa's method plus MacNeal tetrachrome counterstain. Mineralized bone was stained black and collagen type 1 was stained light blue. Bar represents 1 mm. +/+ *ZAS3* WT mice, −/− *ZAS3* KO mice, and +/− heterozygous ZAS3 mice.

Mice (ZAS3 +/+, +/−, and −/−) were generated by heterozygous crosses. Biomechanical analysis (load to fracture; Fx) using three-point bending revealed that the femoral diaphyseal bone strength of adult male *ZAS3−/−* mice (30.88±11.76 N) was significantly greater than those of *ZAS3* WT (17.86±6.13) and their heterozygous littermates (17.98±5.61). Increased bone strength was observed in both male and female *ZAS3−/−* mice (Fig. 1A and Table 1). Furthermore, the outer femoral diameters of *ZAS3−/−* male mice (1.92±0.16 mm) were larger than control mice (1.61±0.08 mm +/+ and 1.70±0.10 mm +/−), whereas the inner diameters (0.55±0.24 mm −/−) were smaller than those of the controls (0.8±0.04 mm +/+, and 0.81±0.10 mm +/−), culminating in significantly larger bone areas in *ZAS3−/−* mice (2.64±0.66 mm^2^ −/−; 1.50±0.19 mm^2^ +/+; and 1.72±0.30 mm^2^ +/−). When force to failure was proportioned to area, there was no significant difference in the stresses among the samples (Table 1), suggesting that the intrinsic properties of the bones were not altered by ZAS3 deficiency. Increase in bone strength and changes in bone dimensions were observed in both male and female ZAS3−/− mice.

**Table 1:** Biomechanical property and dimensions of femur bones.

	ZAS3+/+		ZAS3+/−		ZAS3−/−	
	male	female	male	female	male	female
Load to Fracture (N)						
average	17.86	14.19	17.98	15.73	30.88	34.10
STD	6.13	3.39	5.61	4.33	11.77	12.44
p values			0.96	0.23	0.0012	6.83E-07
Outer dimensions (mm)						
average	1.61	1.50	1.70	1.58	1.92	1.84
STD	0.08	0.087	0.11	0.079	0.16	0.052
p values			0.028	0.24	5.30E-07	0.0027
Inner dimensions (mm)						
average	0.83	0.80	0.82	0.79	0.55	0.635
STD	0.043	0.095	0.10	0.095	0.25	0.033
p values			0.29	0.93	5.6E-05	8.14E-05
AREA (mm^2^)						
average	1.51	1.26	1.72	1.47	2.64	2.34
STD	0.19	0.19	0.31	0.19	0.66	0.15
p values			0.059	0.012	2.34E-06	1.23E-13
Stress to fracture (N/mm^2^)						
average	11.44	11.41	10.64	10.76	11.47	14.58
STD	3.74	2.82	2.45	2.63	2.52	5.05
p values			0.39	0.47	0.76	0.056

Histomorphometric imaging using micro-computed tomography (μCT) showed increased trabecular bone and cortical bone thickness in adult *ZAS3−/−* mice (Fig. 1B and 1C). Additionally, histological analysis of femoral bones with von Kossa's staining showed that the *ZAS3−/−* mice had greater amounts of mineralized bone in the spongiosa, as compared to the WT mice (Fig. 1D). Hence, the increased thickness and mineralization of the cortical and trabecular bone could be additional factors contributing to the increased bone strength of *ZAS3−/−* mice.

### Dynamic femoral bone parameters at different ages of WT and *ZAS3*−/− mice

Histological sections of femurs were prepared to evaluate the bone phenotypes and bone formation parameters in adult *ZAS3−/−* and *ZAS3+/+* control mice, from ages 3 months to 6 months (Table 2). At all ages examined, the volume, number and thickness of the trabecular bones of the *ZAS3−/−* mice were significantly (*p*<0.05) higher than those of sex-matched control littermates. Consequently, the trabecular separation was narrower in *ZAS3*−/− mice. Bone volume fraction and trabecular separation correlated with hardness [22]. Therefore, they could be factors contributing to the increased bone strength in *ZAS3−/−* mice (Fig. 1A). In dynamic measurements, there were no significant differences in the mineral apposition rate between *ZAS3−/−* and control mice. However, the ratio of mineralization surface to bone surface and the bone formation rate, measures of total bone anabolic activity were slightly decreased in the *ZAS3−/−* mice, most notably at 3 months. Whereas a robust phase of bone growth in neonatal *ZAS3−/−* mice, occurring between ages 1 week and 2 weeks, contributed to high bone mass [14], our data suggested that other factors might maintain the high bone mass in adult ZAS3−/− mice.

**Table 2:** Dynamic femoral bone parameters of adult ZAS3 WT and KO mice.

Age months	Genotype	BV/TV (%)	Tr. N. (mm^−1^)	Tr. Sp. (µm)	Tr. Th. (µm)	MAR (mcm/day)	MS/BS (%)	BFR/BS (mcm/day)
6	WT	5.28±1.3	0.76±0.16	1566±218	68.15±12	1.65±0.3	14.69±6.7	27.75±9.2
	KO	58.25±3.5	1.79±0.27	252.68±11	393.94±87	1.63±0.08	13.28±4.7	22.84±6.6
	p values	2.83E-07	0.001127	6E-05	0.002883	0.88509	0.755703	0.441597
5	WT	6.86±3.2	0.82±0.26	1106.10±55	86.54±9.07	1.56±0.36	31.06±8.3	52.98±31.1
	KO	25.02±9.6	1.64±0.37	528.90±291	157.00±33	1.52±0.19	11.17±10.9	30.43±5.46
	p values	0.011718	0.011786	0.028044	0.023907	0.895211	0.027467	0.390637
3	WT	4.85±1.82	0.72±0.18	1798.79±501	64.30±8.09	2.42±0.32	32.45±11.81	111.4±12.5
	KO	53.75±26	1.82±0.38	258.08±114	431.77±33	1.70±0.28	19.98±3.3	58.81±4.9
	p values	0.010885	0.002416	0.00389	0.00118	0.069244	0.018564	0.06453

BV/TV, bone volume/total volume; Tr.N, trabecular number; Tr.Sp, trabecular separation; Tr.Th, trabecular thickness; MAR, mineral apposition rate; MS/BS, mineralization to bone surface; BFR/BS, bone formation rate; CS, cross section; and OC/BS, number of osteoclast/bone surface.

### Femurs of adult *ZAS3−/−* mice have decreased numbers of osteoclast

**Figure 2:**
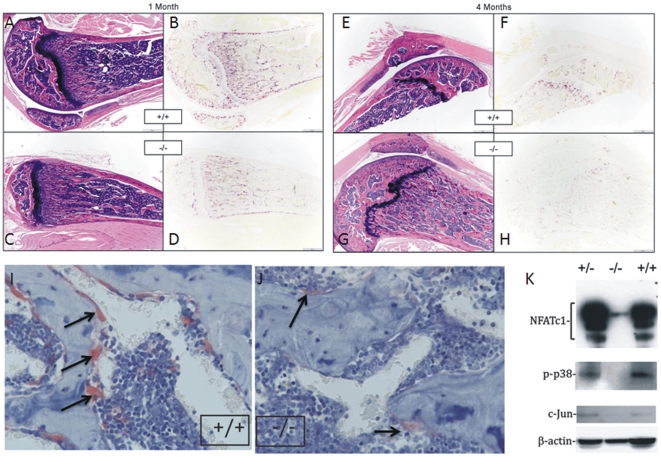
Adult *ZAS3* knockout mice exhibit decreased number of osteoclasts. Sagittal sections of femora from 1-month-old mice (A to D) or 4-month-old mice (E to H) stained with H & E (A, C, E and G) or stained for TRAP (B, D, F and H). (I) and (J) Higher magnifications of femora of 4-month-old mice stained with TRAP. Note that the number of osteoclasts (cells stained red and indicated with arrows) in WT mice (I) was more abundant than that of KO mice (J). (K) Western blot analysis of protein lysates of bone marrow stromal cells with indicated antibodies. +/+ *ZAS3* WT mice, −/− *ZAS3* KO mice, and +/− heterozygous ZAS3 mice.

Adult bone remodeling is a balance between bone formation and bone resorption. Next, we compared the abundance of osteoclasts between *ZAS3−/−* and control mice to determine whether the high bone mass may be due to defective osteoclasts, and hence decrease in bone resorption. Histological bone sections of 1 month and 4 months old mice were stained with H&E or for TRAP, an enzyme highly expressed in osteoclasts (Fig. 2A–H). The intensity and amount of TRAP staining in *ZAS3−/−* mice were less than ZAS3+/+ mice at both ages. The distribution of TRAP was most limited in 4-month-old ZAS3−/− bones (Fig. 2H), at a time when the trabeculae and cortices of the ZAS3−/− bones were approximately twice the thickness of the control ZAS3 +/+ mouse. A higher magnification showed more abundant, larger osteoclasts with more TRAP lining the trabecular bones of 4-month-old WT mice than ZAS3−/− mice (Fig. 2I, J).

Western blot analysis showed that NFATc1, the master transcription factor in osteoclast differentiation, was significantly downregulated in *ZAS3−/−* bone marrow stromal cells comparing to controls (Fig. 2K). Similarly, the activated and phosphorylated forms of p38 and c-Jun were suppressed in *ZAS3−/−* bone marrow cells. These MAP kinases and transcription factors are essential for sRANKL-induced osteoclast differentiation [18], [19], [ and 20 for a review]. The decreased expression of these major osteoclast regulators in *ZAS3−/−* bone marrow stromal cells was in agreement with the histological findings which revealed very few osteoclasts in bones of adult *ZAS3−/−* mice (Fig. 2J). In all, the data showed that adult ZAS3−/− mice had less osteoclasts than control mice. Defective osteoclasts should lead to decreased bone resorption, resulting in mild osteopetrosis in adult *ZAS3−/−* mice.

### Expression and subcellular localizations of ZAS3 associate with osteoclastogenesis of bone marrow precursors

**Figure 3:**
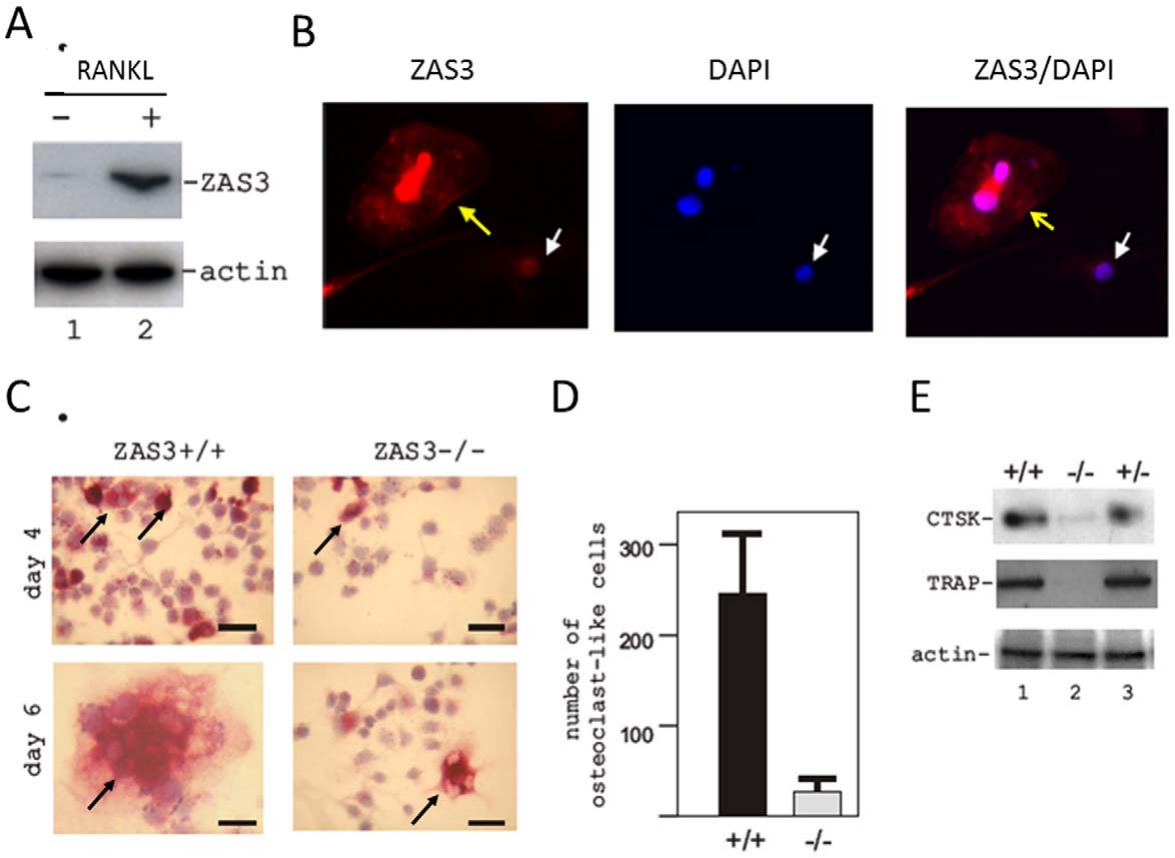
ZAS3 is essential for RANKL-mediated osteoclastogenesis of bone marrow precursors. (A) Western blot analysis of total protein lysates prepared from bone marrow macrophages (BMM) cultured without (−) or with (+) sRANKL (50 ng/ml) for 3 days. Bone marrow aspirates isolated from WT mice were cultured in complete medium with M-CSF (50 ng/ml) for 3 days, and non-adherent cells were harvested and further cultured with M-CSF (50 ng/ml) and sRANKL (50 ng/ml) for 3 days. (B) Immunohistochemical analysis of ZAS3 (red) in bone marrow macrophages (BMM) cultured with M-CSF and sRANKL (50 ng/ml each) at an early phase of osteoclastogenesis. White arrow indicates a mononucleated cell in which ZAS3 shows prominently nuclear localization. Yellow arrow highlights the membrane proximity of ZAS3 in a cell that contained two nuclei and therefore, had undergone the first cell fusion. Nuclei were stained with DAPI (blue). (C) TRAP staining of BMM cultured in the presence of M-CSF and sRANKL (50 ng/ml each). More TRAP+ (cells stained red) and OLC were observed from *ZAS3+/+* than *ZAS3−/−* BMM at day 4 (upper panels) and day 6 (lower panel). At day 6, most *ZAS3+/+* BMM had differentiated into large multinucleated TRAP positive cells while *ZAS3−/−* BMM was inefficient to do so and failed to form large spreading TRAP+ OLC. Scale bar, 100 µm. Data were representatives of 1 out of 3 independent experiments. (D) Bar charts showing the number of TRAP+ multinucleated OLC (more than 3 nuclei) formed in response to sRANKL and M-CSF stimulation (initially plated 5000 cells per well of an 8 well slide). Data were expressed as the mean ± SD from 3 independent experiments. (E) Western blot analysis of protein lysates of BMM cultured with M-CSF and sRANKL for 6 days with indicated antibodies. +/+ *ZAS3* WT mice, −/− *ZAS3* KO mice, and +/− heterozygous ZAS3 mice.

Previously, we have shown that ZAS3 is expressed in macrophages [3]. However, its expression in osteoclast precursors and during osteoclastogenesis has not been determined. Bone marrow stromal cells isolated from *ZAS3+/+* and *ZAS3−/−* mice were differentiated into BMM by incubating with M-CSF (50 ng/ml) for 3 days, and then osteoclast differentiation was induced by the addition of sRANKL. Western blot analysis revealed dramatic increase of ZAS3 proteins after stimulating WT BMM with sRANKL (50 ng/ml) for 3 days (Fig. 3A). In addition, immunohistochemical analysis showed alteration of the subcellular localization of ZAS3 during osteoclastogenesis. In mononuclear BMM, ZAS3 was mainly observed in the nucleus (indicated with a white arrow in Fig. 3B). However, in cells with two nuclei (after the first cell fusion), not only was the nuclear expression of ZAS3 increased dramatically, it was also observed in the cytoplasm. Notably, some cytoplasmic ZAS3 was observed at the cell membrane (indicated with a yellow arrow in Fig. 3B).

Osteoclast differentiation of BMM was monitored by staining for the presence of TRAP and changes in cell morphology (Fig. 3C). After 4 days of sRANKL stimulation, more than 50% of the WT BMM had differentiated into TRAP positive osteoclast-like cells (OLCs) and some with 3 or more nuclei, whereas only 10% of the *ZAS3−/−* BMM were TRAP positive (Fig. 3C, upper panels). At day 6, most WT BMMs had differentiated into giant TRAP positive OLCs, often with more than 10 nuclei (Fig. 3C, lower panels). On the contrary, the number of OLCs derived from *ZAS−/−* BMM was considerably much less, only ∼10% of that of WT (Fig. 3D, *p*<0.05). In addition, such OLCs were generally smaller and with fewer (3 to 4) nuclei (Fig. 3C, lower panels). Consequently, Western blotting showed that after incubation with sRANKL for 4 days, *ZAS3+/+* and *ZAS3+/−* BMMs produced more TRAP and cathepsin K (CTSK), enzymes required for osteoclastic bone resorption, than *ZAS3−/−* BMMs (Fig. 3E). Taken together, the data showed that the expression of ZAS3 was associated with osteoclast differentiation and that ZAS3-deficiency blocked osteoclastogenesis. Therefore, ZAS3 was essential for osteoclast differentiation and function from bone marrow osteoclast precursors.

### Regulation of ZAS3 during osteoclastogenesis of RAW 264.7 cells

**Figure 4:**
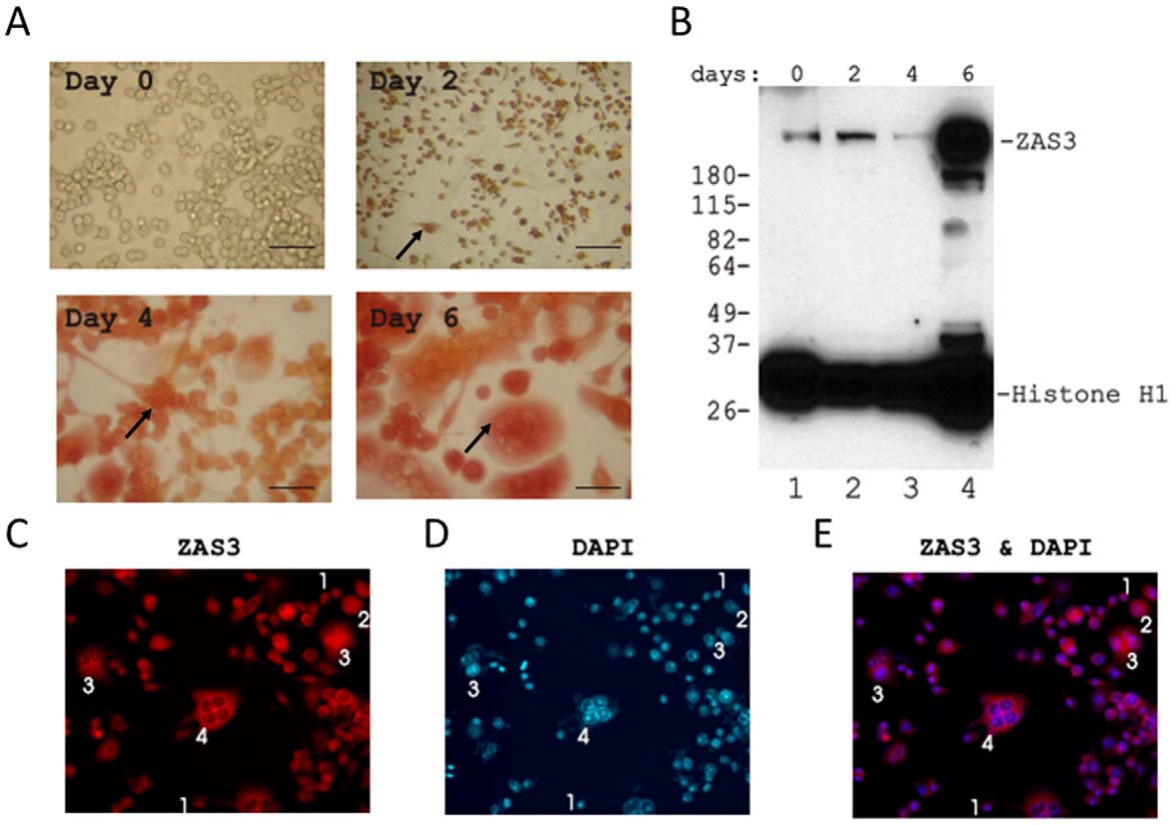
Expression and subcellular localization of ZAS3 associate with osteoclastogenesis of RAW 264.7 preosteoclasts. (A) Morphological changes and increase in TRAP expression of RAW264.7 cells upon sRANKL-mediated osteoclastogenesis. RAW 264.7 cells were cultured for 0, 2, 4 or 6 days and stained for TRAP. Scale bar, 50 µm and arrows show representative TRAP+ cells. (B) Western blot analysis to show the presence of ZAS3 in RAW 264.7 cells at days 0, 2, 4 and 6 incubated with sRANKL (50 ng/ml). The protein filter was also incubated with histone H1 antibodies as a loading control. (C) Immunohistochemical fluorescence microscopy showing the expression of ZAS3 in RAW 264.7 cells at various stages of osteoclast differentiation. Number shown was the number of nucleus or nuclei presence in the cell indicated. During osteoclastogenesis, multinucleated giant cells are progressively formed by cell fusion. Therefore, increasing number of nuclei represented cells were at more advanced stage of osteoclastogenesis.

Next, we examined the expression of ZAS3 in RAW 264.7 preosteoclasts during sRANKL-mediated osteoclastogenesis. Figure 4A shows the morphological changes and increase in TRAP expression during osteoclastogenesis of RAW 264.7 cells. Unstimulated RAW 264.7 cells were small, round, mononuclear, and without detectable TRAP expression. At day 2 of sRANKL (50 ng/ml) stimulation, most cells had assumed a fibroblast-like morphology, developed cytoplasmic extensions, and expressed low levels of TRAP. Thereafter, TRAP expression increased and OLC phenotype progressed rapidly, and at day 6, most cells had differentiated into multinucleated TRAP positive OLCs. Western blot analysis showed significant increase in ZAS3 expression after 6 days of stimulation with sRANKL (Fig. 4B). In addition, immunohistochemical analysis showed that as observed in BMM, the subcellular localization of ZAS3 in RAW 264.7 cells gradually changed during osteoclastogenesis, transiting from the nucleus to the cytoplasm (Fig. 4C–E). Taken together, the expression of ZAS3 was increased and its subcellular localization changed from nuclear to cytoplasmic upon osteoclastogenesis of RAW 264.7 cells. Similar changes in ZAS3 expression that were also observed during osteoclastogenesis of primary bone marrow cells (Fig. 3A, B) suggested such patterns of ZAS3 expression probably associate with differentiation of diverse osteoclast precursors.

### Silencing of ZAS3 suppresses osteoclastogenesis and its forced expression promotes osteoclastogenesis in RAW 264.7 cells

**Figure 5:**
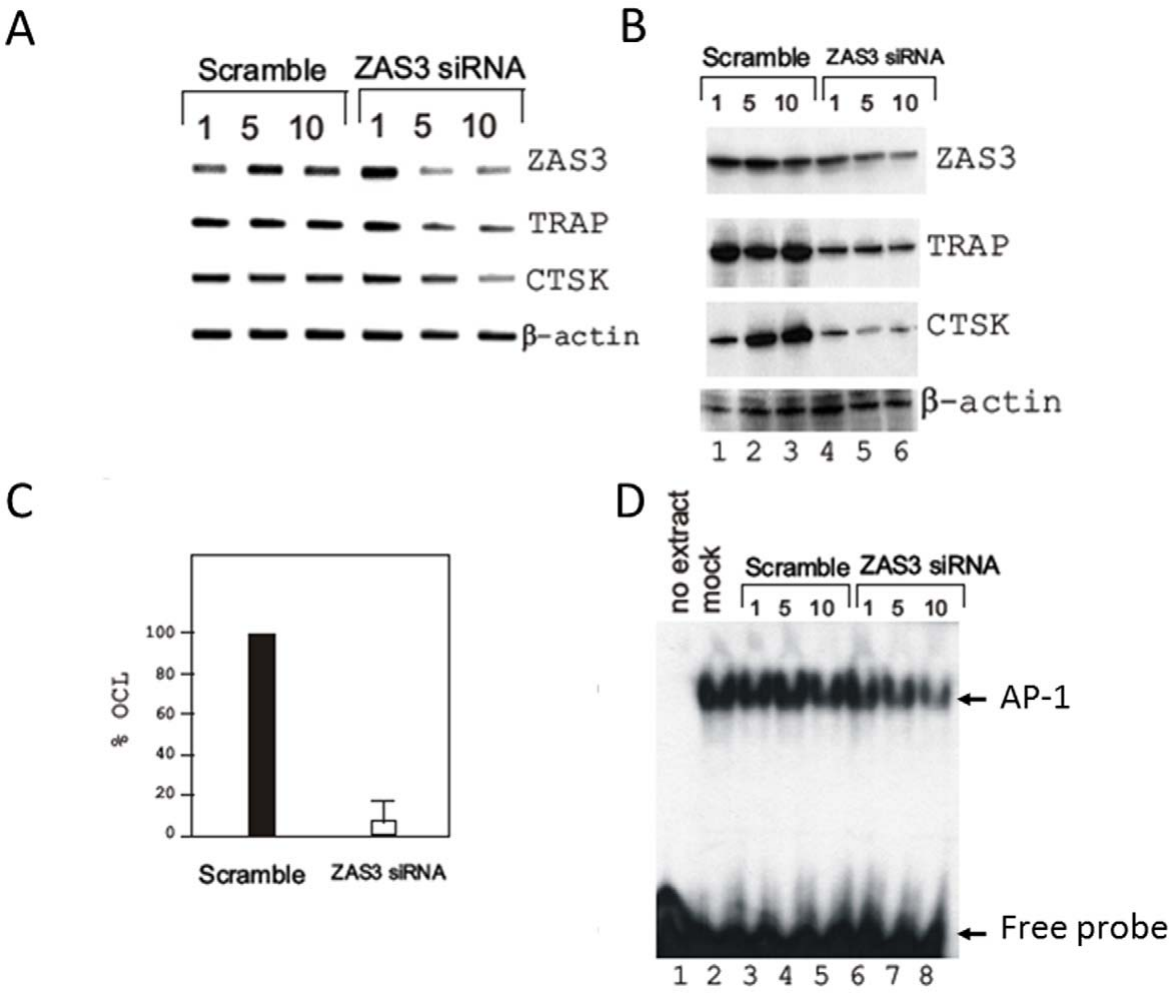
Silencing of ZAS3 inhibits RANKL-mediated osteoclastogenesis in RAW 264.7 cells. RAW264.7 cells were transfected with (1 µM, 5 µM, or 10 µM) scramble siRNA or a pool of four *ZAS3* siRNA and incubated with complete medium supplemented with sRANKL (50 ng/ml). RNA and total protein lysates were prepared 4 days later and analyzed by (A) RT-PCR and (B) Western blot analysis using gene-specific primer sets and antibodies, respectively. (C) The above transfected cells were incubated with sRANKL (50 ng/ml) for 6 days and the numbers of OLC (TRAP positive cells with three or more nuclei) were counted. The percentage of OLC formed by scramble siRNA transfected cells was tentatively assigned as 100. Cells transfected with *ZAS3* siRNA formed relatively much less (10% versus scramble) OLC. Data are expressed as mean ± SD from three independent experiments. (D) RAW 264.7 cells were mock transfected, transfected with scramble siRNA, or with *ZAS3* siRNA, and incubated with sRANKL (50 ng/ml) for 4 days. Subsequently, EMSA was performed with nuclear extracts and ^32^P AP-1 consensus sequences. The amount of siRNA (1, 5, and 10 in micromoles) used in transfection is shown on the top of the lanes.

To delineate the molecular mechanism by which ZAS3 regulates osteoclastogenesis, we silenced ZAS3 expression in RAW 264.7 cells by transfecting a pool of four short interference RNAs (siRNAs) that targeted different protein coding regions of the ZAS3 transcripts and determined how that might affect sRANKL-induced osteoclastogenesis. Four days after transfection and incubation with sRANKL (50 ng/ml), the relative levels of ZAS3 transcripts and proteins in RAW 264.7 cells were significantly decreased by *ZAS3* siRNA (at 5 µM and 10 µM concentrations) than those of scramble controls (Fig. 5A, B). Notably, the expression of TRAP and CTSK was also decreased in the ZAS3 silenced samples, suggesting a positive relationship between the expression of ZAS3 and these two crucial osteoclast-associated genes. As a negative control, the expression of β-actin was not affected by ZAS3 silencing (Fig. 5A, B). In addition, the number of sRANKL-induced OLC formed at day 6 was significantly reduced in the ZAS3 silenced cells, as compared to scramble siRNA transfected controls (Fig. 5C). Because c-Jun, a component of AP-1 that is important for osteoclastogenesis, was downregulated in *ZAS3−/−* bone marrow cells (Fig. 2B), we performed EMSA and showed that nuclear extracts prepared from ZAS3 silenced RAW 264.7 cells yielded significantly less AP-1-protein complexes than control samples (Fig. 5D). Hence, the data showed that silencing ZAS3 in RAW 264.7 cells inhibited osteoclastogenesis and the expression of important osteoclast-associated gene products, TRAP, CTSK and AP-1.

**Figure 6:**
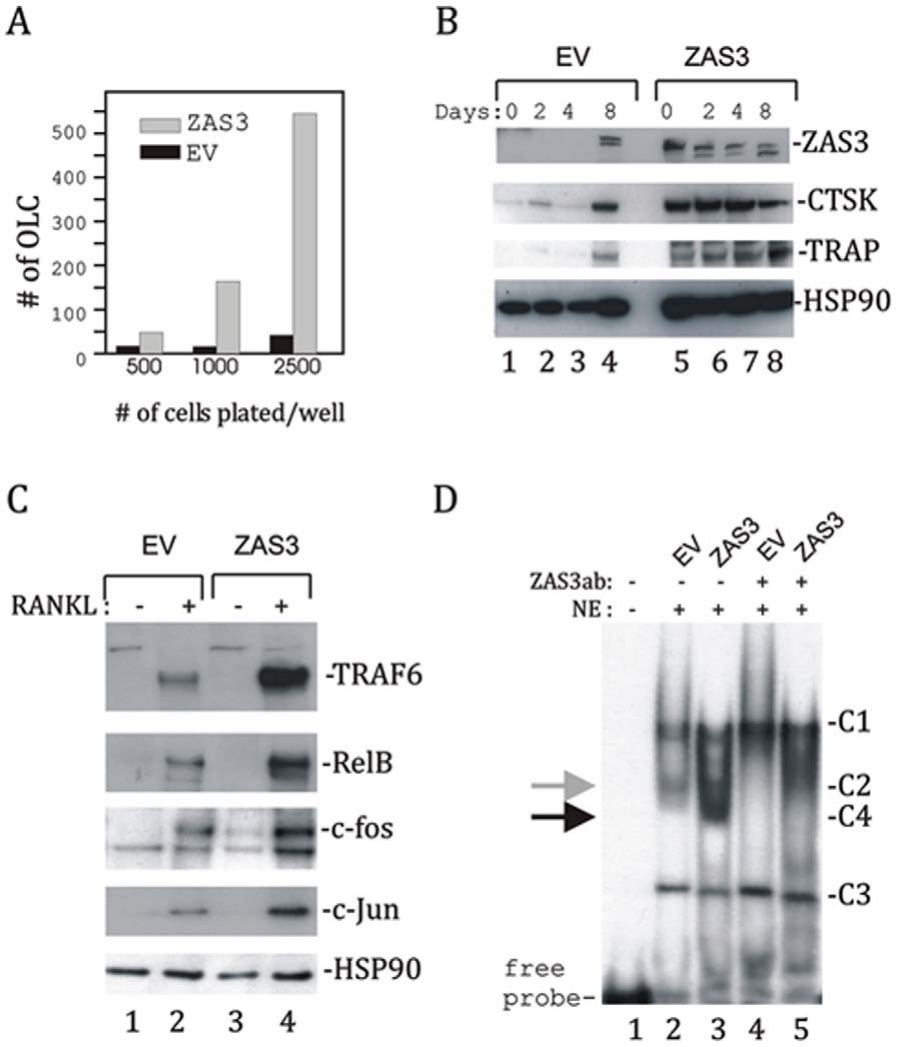
ZAS3 promotes RANKL-mediated osteoclastogenesis and expression of osteoclast-associated genes. RAW264.7 cells (from 500 to 2500 cells) were stably transfected with the empty vector (EV) or the ZAS3 expression construct. (A) Indicated cells were plated onto wells of a 24-well plate and cultured in complete medium supplemented with sRANKL (50 ng/ml) for 6 days, and stained for TRAP. TRAP+ cells with 3 or more nucleic were considered as OLC and counted. Bar charts show the numbers of OLC. (B) Indicated cells were incubated with sRANKL (50 ng/ml) for 0, 2, 4, or 8 days. The induction of ZAS3, TRAP and CTSK (osteogenic markers), and HSP90 (loading control) was examined in total cell lysates by Western blot analysis. (C) Indicated cells were incubated with sRANKL (50 ng/ml) for 6 days and the induction of TRAF-6, RelB, c-Fos, and c-Jun was analyzed by Western blot analysis. (D) EMSA was performed with ^32^P-GTTCTGGGGAAGTCCAGTGCTCACATGACC DNA probe corresponding to the mouse TRAP gene enhancer (ZAS3 binding site is shown in underline) and nuclear extracts prepared from indicated cells. The major DNA-protein complexes are designed C1 to C4. Complex C4 (indicated with a black arrow) was observed only in the ZAS3 transfected samples (lane 3). The formation of C2 (indicated with a grey arrow) and C4 was abolished by the addition of ZAS3 antibodies to the binding reactions (lanes 4 and 5).

Next we determined whether overexpression of ZAS3 would promote osteoclastogenesis. RAW 264.7 cells were stably transfected with a mammalian expression vector encoding a nearly complete ZAS3 protein (from amino acids 106 to 2013 of the 2384 residues). Because osteoclasts are formed by cell fusion, cell density is also critical for the formation of multinucleated OLC. Therefore, three titrations of cells (500, 1000, and 2500 cells per well) were cultured with sRANKL for 6 days. In all cases, the numbers of OLC formed by ZAS3 transfectants were higher, by 2–10 fold, than control cells transfected with the empty vector (Fig. 6A). Western blot analysis showed that endogenous ZAS3 expression in parental RAW 264.7 was low and was significantly induced by sRANKL at day 8 (Fig. 6B). On the other hand, the expression of recombinant ZAS3 proteins in the ZAS3 transfectants was readily detectable even in unstimulated cells. Recombinant ZAS3 was not induced by sRANKL because its expression was under the control of a heterologous CMV promoter. In addition, the expression of CTSK and TRAP was generally much higher in ZAS3 overexpressing cells, supporting our notion that ZAS3 positively regulates these osteoclastic genes. As a control of protein loading, Western blot analysis was performed with heat shock protein 90 (HSP90) antibodies. Consequently, we also examined the expression of several important signaling molecules and transcription factors in RANK signaling, and found that ZAS3 further enhanced the RANKL-induced expression of TRAF6, RelB, c-Fos and c-Jun (Fig. 6C).

The expression of TRAP was positively associated with of ZAS3. TRAP expression was higher in ZAS3 overexpressing RAW 264.7 cells (Fig. 6B), and was lower in ZAS3−/− BMM (Fig. 3C & E) and in ZAS3 silenced RAW 264.7 cells (Fig. 5B). Therefore, we performed DNA-protein interaction analysis of ZAS3 and the *TRAP* gene regulatory element to evaluate whether ZAS3 regulates transcription of *TRAP*. The proximal enhancer of *TRAP*, located at nucleotides 26 to 55 upstream of the transcription start site containing binding sites for MITF, PU.1 and EOS, has been characterized [23]. An inspection of that enhancer sequence revealed a putative ZAS3 binding site, GGGGNNNNCC [2]. In EMSA, nuclear extracts of RAW 264.7 control and ^32^P-labeled TRAP enhancer sequence yielded three major DNA-protein complexes, designated as C1, C2 and C3 (Fig. 6D, lane 2). Nuclear extracts prepared from ZAS3 overexpressing cells yielded an additional complex, C4, with a gel mobility slightly faster than C2 (lane 3 and indicated with a black arrow). To determine whether those DNA-protein complexes contained ZAS3, antibody supershift assays were performed. The addition of ZAS3 antibodies to the binding reaction abolished the formation of C2 and C4 complexes, suggesting that they contained ZAS3. Most likely, complex C2 contained endogenous ZAS3 protein, whereas complex C4 contained recombinant ZAS3 protein. Taken together, these experiments demonstrated that ZAS3 binds to the *TRAP* enhancer. Most likely ZAS3 serves a transcriptional function to promote expression of osteoclastic genes and osteoclastogenesis.

### ZAS3 associates with TRAF6

**Figure 7:**
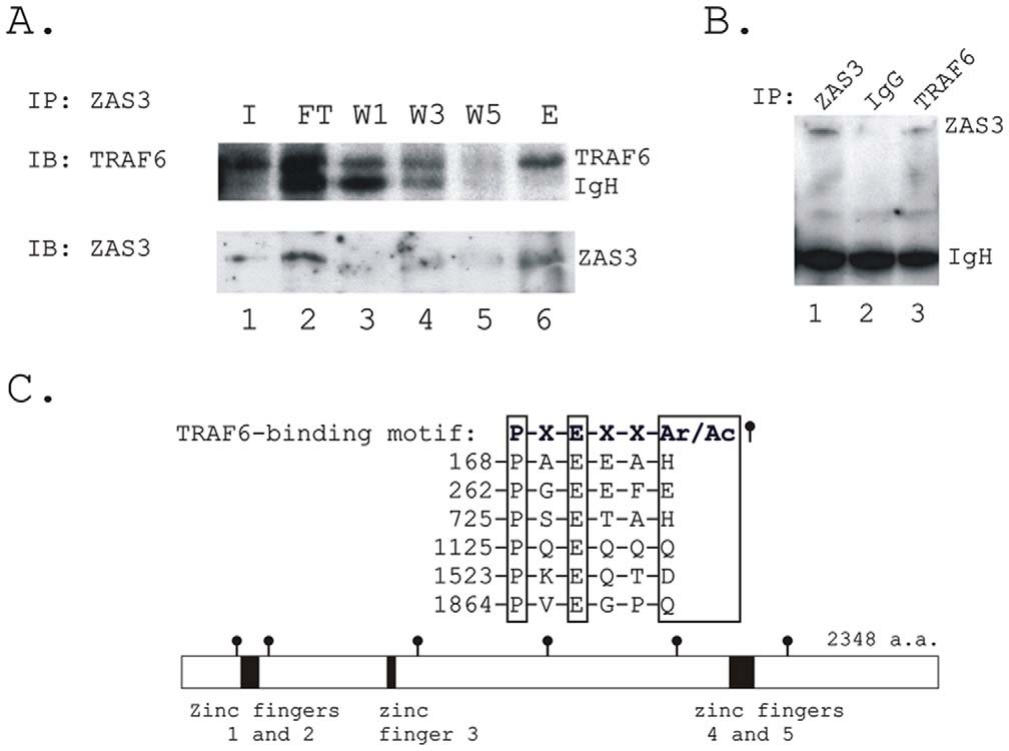
ZAS3 associates with TRAF6. (A) RAW264.7 cells were incubated with sRANKL (50 ng/ml) for 2 days and immunoprecipitation (IP) experiment was performed. Protein lysates were incubated with ZAS3 antibodies, washed excessively, and input (I), flow through (FT); wash 1 (W1), wash 3 (W3), wash 5 (W5), and eluates of immunoprecipitated proteins (E) were subjected to Western blot analysis. Immunoblottings (IB) were performed with TRAF6 or ZAS3 antibodies. (B) RAW 264.7 cells were treated and IP was performed as described in (A). Protein lysates were IP with ZAS3 antibodies, TRAF6 antibodies or control IgGs as indicated on the top of each lane, and Western blot analysis was performed with ZAS3 antibodies to show the presence of ZAS3 in both the immunoprecipitates of ZAS3 and TRAF6 antibodies samples, and its absence in control IgG sample. (C) Putative TRAF6 binding sites in the ZAS3 protein. Top, amino acid sequence alignment showing the TRAF6-binding motif [25] matched with 6 regions in the ZAS3 protein. Ar, an aromatic and Ac an acidic amino acid residue. Bottom, a diagram showing the locations of the six putative TRAF6-binding sites in the ZAS3 protein. The localizations of the zinc fingers in the ZAS3 protein were also shown.

Finally, we examined the cytoplasmic function of ZAS3. Based on the membrane proximity of ZAS3 during osteoclastogenesis (shown with a yellow arrow in Fig. 3B) and the ability of ZAS3 to associate with TRAF2 [12], we determined whether ZAS3 could associate with TRAF6, the major member of the TRAF protein family involved in osteoclastogenesis [24]. RAW264.7 cells were stimulated with sRANKL (50 ng/ml) for 2 days, and immunoprecipitation (IP) experiments of total cell lysates were performed with ZAS3, TRAF6 or control IgG antibodies. The presence of TRAF6 in the eluate of the IP experiments using ZAS3 antibodies and *vice versa*, suggested that ZAS3 interacted with TRAF6 (Fig. 7A, B). Furthermore, initial IP experiments using unstimulated cells did not detect such protein-protein interactions, suggesting that the interaction of ZAS3 and TRAF6 was induced by RANKL. ZAS3 could associate directly with TRAF6 or via other adaptor molecules. In support of the former notion, an inspection of the ZAS3 protein sequence revealed six putative TRAF6-binding motifs [Pro-X-Glu-X-X-(Ar/Ac), Ar an aromatic and Ac an acidic residue] in the ZAS3 protein [25]. Figure 7C shows their amino acid sequence alignment and the locations of the six TRAF6 binding motifs in the ZAS3 protein. In all, the results of the co-IP experiments and the presence of putative TRAF6 binding site in the ZAS3 protein suggested that ZAS3 may directly associate with TRAF6.

## Discussion

Present findings demonstrate that ZAS3, a large zinc finger protein, is a critical regulator of bone resorption and controls bone mass via activation of osteoclasts. This is evident from the observations that the bones of adult *ZAS3−/−* mice are thicker and more resistant to fractures. The biomechanical properties contributing to the increased bone strength in *ZAS3−/−* mice likely are the summation of several factors, including increased cortical bone thickness, more mineralization, increased trabecular bone volume and number, and decreased in trabecular separation. In bone, increase in the osteoblast activity contributes to increase in bone formation and bone mass in neonatal *ZAS3−/−* mice [14]. Here we show that osteoclasts in adult *ZAS3−/−* mice are defective, which should lead to decrease in bone resorption, and consequently, higher bone mass. The decrease in the number of osteoclast may lead to decreased bone resorption, and may augment the deposition of more densely mineralized bone, with reduced porosity and the temporary deficit of bone that occurs between bone resorption and formation. Together with the role of ZAS3 in osteoblasts [14], our findings suggest that ZAS3 also regulates osteoclast differentiation and thus must be critical in postnatal skeletal remodeling.

Orthologous ZAS proteins from humans to lower eukaryotes have been shown to be involved in skeletal development. For examples, sma-9 in nematodes regulates pathways of defective body length-1 (DBL-1) [27] and schnurri (Shn) in Drosophila regulates decapentaplegic (dpp), the homologue of vertebrate BMPs [28]. BMPs play key roles in both osteoblast proliferation and differentiation, as well as in osteoclast differentiation [reviewed in 29]. In mice, gene knockout experiments show that the ZAS proteins are required for both osteoblast and osteoclast development [14]–[16], and together this protein family might orchestrate postnatal skeletal development. Although the generation of *ZAS1* knockout mice has not been reported, *ZAS1* has also been shown to inhibit the expression of the type II collagen, a major extracellular matrix protein in cartilage [6]. Recently, double knockout mice for *ZAS2* (*Shn2*) and *ZAS3* (*Shn3*) have been generated, and they exhibit impaired growth plate maturation during endochondral ossification and massively elevated trabecular bone formation [17]. Earlier findings that ZAS3 augments osteoblast bone formation together with our findings that ZAS3 is crucial for osteoclast bone resorption suggest that ZAS3 plays an integral role in adult bone remodeling.

We have observed that the lack of ZAS3 in bone marrow precursors and silencing of ZAS3 in preosteoclast directly suppress RANKL-mediated osteoclast differentiation. Conversely, forced expression of ZAS3 promotes osteoclastic differentiation. These observations along with reduction of osteoclasts in bones of ZAS3−/− mice strongly point to an essential role of ZAS3 in osteoclastogenesis. We further confirmed the role of ZAS3 in sRANKL mediated induction of osteoclast differentiation by demonstrating the ability of ZAS3 to bind to the TRAP promoter and associates with TRAF6. These experiments suggest that ZAS3 might have multiple functions in regulating osteoclastogenesis. It serves as a transcription factor to regulate osteoclast-associated gene transcription and as a signaling molecule to regulate the RANK/RANKL signaling pathway. Our notion can be further supported by the increased expression of ZAS3 and its presence in both cytoplasmic and nuclear compartments during RANKL-mediated osteoclastogenesis of primary bone marrow precursors as well as RAW 264.7 preosteoclasts. The similar pattern of gene expression of ZAS3 in both cells suggests this phenomenon could be a general mechanism by which ZAS3 regulates osteoclast differentiation. Many components in RANK signaling central to osteoclastogenesis, including NFATc1, phospho-p38 and c-Jun, are downregulated in the absence of ZAS3. On the other hand, the expression of key osteoclast-associated molecules, such as enzymes (CTSK and TRAP), signaling molecules (TRAF6 and p-p38) and transcription factors (RelB, c-Fos and c-Jun, and NFATc1) are enhanced by ZAS3 upon RANKL-induction. The data suggest that ZAS3 is important in multiple aspects of osteoclast formation and activity, including differentiation, survival and bone resorptive functions.

**Figure 8:**
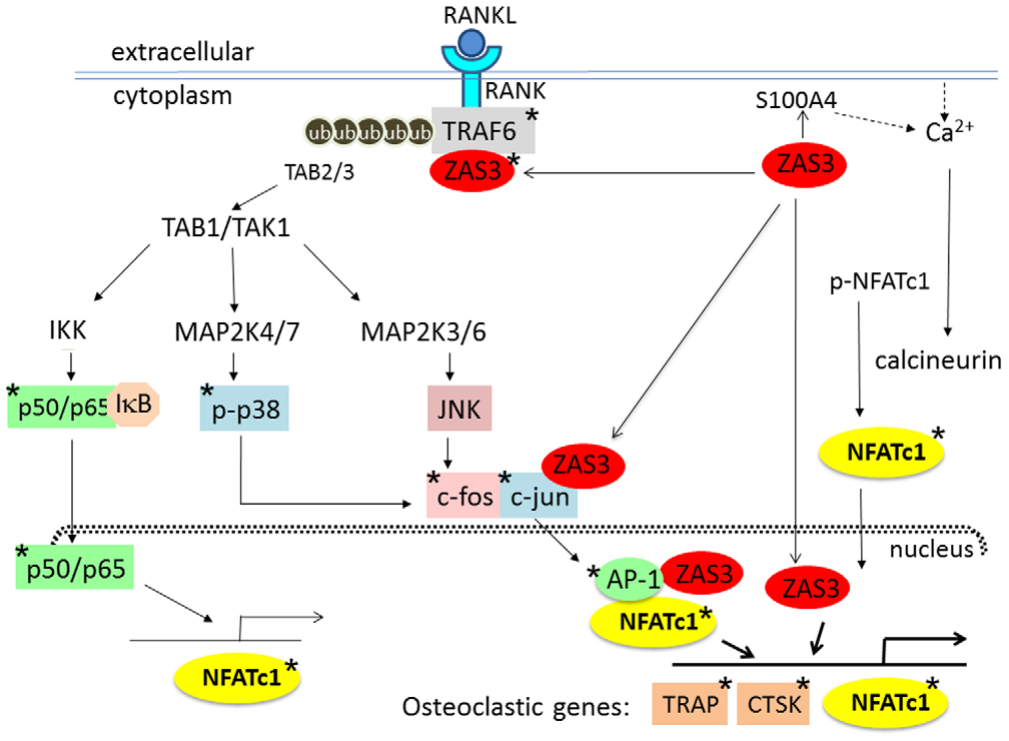
Molecular targets of ZAS3 in RANK signaling. Shown is a schematic diagram depicting the multiple targets of ZAS3 involved in the regulation of RANK signaling, important for osteoclastogenic differentiation and function. Binding of RANKL to RANK activates cytoplasmic ZAS3 that (i) induces expression of TRAF6 and association of ZAS3 and TRAF6 leading to the recruitment of TGF-β-activated kinase 1 (TAK1) binding protein 2/3 (TAB2/3) to polyubiquitinated TRAF6, which in turn activates TAB1/TAK1, inhibitor of NF-kB alpha (IkBα) kinase (IKK) complexes, and nuclear translocation of NF-kB p50/p65; (ii) Simultaneously, ZAS3 activates mitogen-activated protein kinases to activate the transcription factor AP-1 via phosphorylation of p38 and assembly of c-Jun/Fos; (iii) ZAS3 associates with c-Jun to activate AP1 [13]; (iv) ZAS3 itself can act as a transcription factor, i.e., it translocates into the nucleus and binds to gene regulatory elements to activate transcription of osteoclast-associated genes, such as TRAP and CTSK, and probably NFATc1; and (iv) through the activation of NF-kB, ZAS3 also activates NFATc1 that is shown to integrate sRANKL signaling in the terminal differentiation of osteoclasts [30]. Additionally, ZAS3 mobilizes intracellular calcium, probably through one of its target genes, the calcium binding protein *S100A4/mts1* [4], to activate calcineurin causing the dephosphorylation and nuclear translocation of NFATc1. ZAS3 individually, or in a transcriptional complex in conjunction with NTAFc1 and NF-kB through association with AP-1 drives the transcription program for osteoclast differentiation. Important signaling molecules, transcription factors, or enzymes involved in RANK signaling whose expression levels or protein-protein interactions shown here to be regulated by ZAS3 are indicated with asterisks.

In conclusion, our studies demonstrate that ZAS3 is a crucial regulator of bone mass, is required for bone remodeling, and regulates bone resorption via induction of osteoclast differentiation and function. The significance of ZAS3 in RANK signaling is evident from the phenotype of adult ZAS3 deficient mice, which have thicker bones and fewer osteoclasts. Using RAW 264.7 preosteoclast cells, we further showed that forced expression of ZAS3 promotes osteoclastogenesis, while silencing of ZAS3 results in a significant reduction of RANKL's ability to induce osteoclastogenesis and expression of osteoclastic genes, including NFATc1, the master regulator of osteoclast differentiation [31], [32]. Based on the expression and dynamic localization of ZAS3 in the cytoplasm and nucleus, and its ability to associate with specific proteins (such as TRAF6 shown here and c-Jun [13]) and bind to *cis*-acting gene regulatory DNA elements (the TRAP promoter shown herein), we propose that ZAS3 should have multiple cytoplasmic and nuclear targets to modulate osteoclastogenesis via the RANK signaling cascade either directly or via activation of NFATc1 (Fig. 8). Starting from the RANK receptor proximate adaptor protein TRAF6, overexpression of ZAS3 leads to the upregulation of TRAF6 which could intensify RANK signaling. In addition, the fact that ZAS3 also associates with an E3 ubiquitin ligase, WWP1, tempts us to speculate that if cytoplasmic ZAS3 form a complex with TRAF6 and WWP1 that could facilitate activation of TRAF6 through polyubiquitination [14]. Polyubiquitinated TRAF6 recruits TAB2/3, which in turn may activate TAB1/TAK1 and ultimately NFATc1 via: (i) assembly of the IKK complex, ubiquitination and degradation of IkB, and translocation of p50/p65 into the nucleus to activate the transcription of NFATc1 [31]; (ii) activation of AP1 via MAP kinase signaling cascades to induce phosphorylation of p38 and assembly of c-Jun/c-Fos, which is shown to be critical for NFATc1 activation and autoactivation [18], [19], [30]; and (iii) mobilization of intracellular calcium, probably through the calcium binding protein S100A4/mts1 that is a ZAS3 target gene [4], which results in the activation of calcineurin that dephosphorylates NFATc1 and allowing its translocation into the nucleus. Furthermore, when translocated into the nucleus, ZAS3 binds to the promoter regions of osteoclast-associated genes, such as TRAP, or as a part of the transcriptional complex that contains NFATc1 and AP-1 for the activation of osteoclast-specific genes, such as CTSK, TRAP, and NFATc1. Based on these findings, ZAS3 appears to be the central regulator of osteoclastogenesis, warranting further delineation of its role in diseases where skeleton is compromised due to osteoclast hyperactivity.

## Materials and Methods

### Mice, reagents and antibodies

The generation of ZAS3 knockout mice has been described [15]. Mice, +/+, +/− and −/−, used in this study were generated by +/− crosses, and most experiments were performed with sex-matched littermates. Use and care of mice in this study were approved by the Ohio State University Institutional Animal Care and Use Committee (Protocol Number 2008A0034). Recombinant murine M-CSF and sRANKL were purchased (Peprotech, NJ). ZAS3 antibodies have been described [4], and other antibodies were purchased from Santa Cruz Biotechnology, CA.

### Bone histomorphometry and mechanical testing

Biomechanical properties of femurs were evaluated by three-point bending test on a servo hydraulic material test frame using an MTS 858 Bionix Testing Machine (MTS Systems Corporation, MN) at 1 mm/min with continuous recording of load until failure. After mechanical testing, cross-sectional geometry [inner diameter (I) and outer diameter (O)] at mid-diaphysis were obtained using a hand-held digital caliper, and area was calculated [area = π(O^2^−I^2^)/4]. Histomorphometric analyses were performed by The Bone Histomorphometry Core Laboratory of the University of Texas, MD Anderson Cancer Center, and The OSU Mineralized Tissue Mechanics Laboratory. Consistent results were obtained from both facilities. For bone formation analysis, mice were injected intraperitoneally with calcein (Sigma-Aldrich, MO) (20 mg/kg) on day 1 and day 4, and bone harvested on day 6.

### Osteoclast cultures and TRAP staining

Bone marrow stromal cells of 4- to 10-week-old mice were flushed from femurs and cultured with complete medium supplemented with M-CSF (50 ng/ml) for 3 days for macrophage differentiation. Non-adherent cells were further cultured with the addition of sRANKL (50 ng/ml) to induce osteoclast formation. TRAP staining of femurs was performed by the OSU Comprehensive Cancer Center Comparative Pathology & Mouse Phenotyping Shared Resource, and of cells was performed using a leukocyte acid phosphatase kit (Sigma-Aldrich, MO). TRAP-positive cells containing three or more nuclei were considered as OLC and counted manually under a light microscope.

### Transfection of RAW 264.7 cells

A mammalian expression construct encoding amino acids 106 to 2013 of the ZAS3 protein has been described [11]. On-target^plus^ Smart pool siRNAs containing a mixture of 4 oligonucleotides with potential for mouse ZAS3 (HIVEP3) mRNA destruction by RISC complexes was used to silence ZAS3 (Thermo Scientific, MA). SiRNA oligonucleotides or plasmid constructs were introduced into RAW 264.7 cells (3×10^6^ cells) by Nucleofector transfection Kit (Amaxa, MD) according to the manufacturer's protocol. A mixture of 4 scrambled siRNAs was used as the negative control. Following transfection, cells were grown in RPMI containing 10% fetal bovine serum, M-CSF (50 ng/ml), and sRANKL (50 ng/ml) on microscopic slides or 6 cm tissue culture plates. Cells were harvested 4 days later and analyzed for the ZAS3 knockdown by RT-PCR and Western blot analysis. Cells stably transfected with the ZAS3 constructs were selected with G418 (0.4 mg/ml) and incorporation of the constructs was validated by PCR of genomic DNA.

### Western blot analysis, immunoprecipitation, and immunohistochemistry

Western blotting and immunohistochemistry of cells on slides were performed as described [33], [34]. For immunoprecipitation, protein lysates were prepared from RAW 264.7 cells incubated with sRANKL (50 ng/ml) for 2 days. Target proteins were immunoprecipitated by incubating total lysates with the relevant antibodies using Catch and Release v2.0 Reversible Immunoprecipitation System (Millipore, Temecula, CA) with the following modifications. Cell lysate, antibodies and antibody capture affinity ligand were incubated at 4°C for 16 hours, and the column was washed with wash buffers for 5 times.

### RNA analysis

RNA was prepared using TRIzol reagent and reverse transcribed by Superscript III reverse transcriptase (Invitrogen, CA). Standard PCR was conducted and β-actin levels were used as controls [35]. Sequences of PCR primers: TRAP-F: 5′-TGACCACAACCTGCAGTATC-3′; TRAP-R: 5′CCCAGGGAGTCCTCAGATCC-3′; CTSK-F: 5′-AAGTGGTTCAGAAGATGACGGGAC-3′; CTSK-R: 5′-TCTTCAGAGTCAATGCCT CCGTTC-3′; β-Actin-F: 5′-CCGGGACCTGACAGACTACC; β-Actin-R: 5′-TGCCACAGGATTCCATACCC; ZAS3-F: 5′-TCCTGAGATCTAAGCAGAAG-3′ and ZAS3-R: 5′-GAGTCTGAGTCAGAGTCCTC-3′.

### EMSA and antibody gel supershift assays

Nuclear extracts was prepared with a Nuclear Extract Kit according to manufacturer instructions (Active Motif, Carlsbad, CA). EMSA with nuclear extracts (2–5 µg) and ^32^P-labeled DNA probes were performed as described [36]. The sense strand oligonucleotide, representing the mouse TRAP proximal sequences, was 5′-TTCTGGGGAAGTCCAGTGCTCACATGACCCA-3′, and AP-1 was 5′-CCGGATTGACTCACGCTTCCAC-3′. Complementary oligonucleotides were annealed and labeled with ^32^P-dCTP and Klenow. In antibody gel supershift assays, after incubating protein and DNA probes for 10 minutes, ZAS3 antibodies were added and the binding reactions were incubated for another 15 minutes before gel loading.

### Statistics

Data were expressed as mean ± SD. Statistical significance was determined by 2-tailed Student's *t* test using Microsoft Excel. *P*<0.05 was considered statistically significant.

## References

[1] Wu LC (2002). ZAS: C_2_H_2_ zinc finger proteins involved in growth and development.. Gene Expr.

[2] Allen CE, Wu LC, Iuchi Shiro, Kuldell Natalie (2004). ZAS zinc finger proteins: the other kappa-B-binding protein family.. Zinc finger proteins from atomic contact to cellular function.

[3] Wu LC, Mak CH, Dear N, Boehm T, Foroni L (1993). Molecular cloning of a zinc finger protein which binds to the heptamer of the signal sequence for V(D)J recombination.. Nucleic Acids Res.

[4] Hjelmsoe I, Allen CE, Cohn MA, Tulchinsky EM, Wu LC (2000). The kappa B and V(D)J Recombination Signal Sequence Binding Protein KRC Regulates Transcription of the Mouse Metastasis-associated Gene S100A4/mts1.. J Biol Chem.

[5] Fan CM, Maniatis T (1990). A DNA-binding protein containing two widely separated zinc finger motifs that recognize the same DNA sequence.. Genes Dev.

[6] Yang X, Li J, Qin H, Yang H, Li J (2005). Mint represses transactivation of the type II collagen gene enhancer through interaction with alpha A-crystallin-binding protein 1.. J Biol Chem.

[7] Dorflinger U, Pscherer A, Moser M, Rummele P, Schule R (1999). Activation of somatostatin receptor II expression by transcription factors MIBP1 and SEF-2 in the murine brain.. Mol Cell Biol.

[8] Makino R, Akiyama K, Yasuda J, Mashiyama S, Honda S (1994). Cloning and characterization of a c-myc intron binding protein (MIBP1).. Nucleic Acids Res.

[9] Jin W, Takagi T, Kanesashi SN, Kurahashi T, Nomura T (2006). Schnurri-2 controls BMP-dependent adipogenesis via interaction with Smad proteins.. Dev Cell.

[10] Shukla A, Malik M, Cataisson C, Ho Y, Friesen T (2009). TGF-beta signalling is regulated by Schnurri-2-dependent nuclear translocation of CLIC4 and consequent stabilization of phospho-Smad2 and 3.. Nat Cell Biol.

[11] Yakovich AJ, Jiang B, Allen CE, Du J, Wu LC (2011). ZAS3 accentuates transforming growth factor beta signaling in epithelial cells.. Cell Signal.

[12] Oukka M, Kim ST, Lugo G, Sun J, Wu LC (2002). A mammalian homolog of Drosophila schnurri, KRC, regulates TNF receptor-driven responses and interacts with TRAF2.. Mol Cell.

[13] Oukka M, Wein MN, Glimcher LH (2004). Schnurri-3 (KRC) interacts with c-Jun to regulate the IL-2 gene in T cells.. J Exp Med.

[14] Jones DC, Wein MN, Oukka M, Hofstaetter JG, Glimcher MJ (2006). Regulation of adult bone mass by the zinc finger adapter protein Schnurri-3.. Science.

[15] Allen CE, Richards J, Muthusamy N, Auer H, Liu Y (2007). Disruption of ZAS3 in mice alters NF-kappaB and AP-1 DNA binding and T-cell development.. Gene Expr.

[16] Saita Y, Takagi T, Kitahara K, Usui M, Ezura Y (2007). Lack of schnurri-2 expression associates with reduced bone remodeling and osteopenia.. J Biol Chem.

[17] Jones DC, Schweitzer MN, Wein M, Sigrist K, Takagi T (2010). Uncoupling of growth plate maturation and bone formation in mice lacking both Schnurri-2 and Schnurri-3.. Proc Natl Acad Sci U S A.

[18] David JP, Sabapathy K, Hoffmann O, Idarraga MH, Wagner EF (2002). JNK1 modulates osteoclastogenesis through both c-Jun phosphorylation-dependent and -independent mechanisms.. J Cell Sci.

[19] Ikeda F, Nishimura R, Matsubara T, Tanaka S, Inoue J (2004). Critical roles of c-Jun signaling in regulation of NFAT family and sRANKL-regulated osteoclast differentiation.. J Clin Invest.

[20] Novack DV, Teitelbaum SL (2008). The osteoclast: friend or foe?. Annu Rev Pathol.

[21] Matsumoto M, Sudo T, Saito T, Osada H, Tsujimoto M (2000). Involvement of p38 mitogen-activated protein kinase signaling pathway in osteoclastogenesis mediated by receptor activator of NF-kappa B ligand (sRANKL).. J Biol Chem.

[22] Nakabayashi Y, Wevers HW, Cooke TD, Griffin M (1994). Bone strength and histomorphometry of the distal femur.. J Arthroplasty.

[23] Hu R, Sharma SM, Bronisz A, Srinivasan R, Sankar U (2007). Eos, MITF, and PU.1 recruit corepressors to osteoclast-specific genes in committed myeloid progenitors.. Mol Cell Biol.

[24] Lomaga MA, Yeh WC, Sarosi I, Duncan GS, Furlonger C (1999). TRAF6 deficiency results in osteopetrosis and defective interleukin-1, CD40, and LPS signaling.. Genes Dev.

[25] Ye H, Arron JR, Lamothe B, Cirilli M, Kobayashi T (2002). Distinct molecular mechanism for initiating TRAF6 signalling.. Nature.

[26] Suda T, Takahashi N, Udagawa N, Jimi E, Gillespie MT (1999). Modulation of osteoclast differentiation and function by the new members of the tumor necrosis factor receptor and ligand families.. Endocr Rev.

[27] Liang J, Lints R, Foehr ML, Tokarz R, Yu L (2003). The Caenorhabditis elegans schnurri homolog sma-9 mediates stage- and cell type-specific responses to DBL-1 BMP-related signaling.. Development.

[28] Dai H, Hogan C, Gopalakrishnan B, Torres-Vazquez J, Nguyen M (2000). The zinc finger protein schnurri acts as a Smad partner in mediating the transcriptional response to decapentaplegic.. Dev Biol.

[29] Baldridge D, Shchelochkov O, Kelley B, Lee B (2010). Signaling pathways in human skeletal dysplasias.. Annu Rev Genomics Hum Genet.

[30] Asagiri M, Sato K, Usami T, Ochi S, Nishina H (2005). Autoamplification of NFATc1 expression determines its essential role in bone homeostasis.. J Exp Med.

[31] Takayanagi H, Kim S, Koga T, Nishina H, Isshiki M (2002). Induction and activation of the transcription factor NFATc1 (NFAT2) integrate sRANKL signaling in terminal differentiation of osteoclasts.. Dev Cell.

[32] Hirotani H, Touhy N, Woo JT, Stern PH, Clipstone NA (2004). The calcineurin/NFAT signaling pathway regulates osteoclastogenesis in RAW 264.7 cells.. J Biol Chem.

[33] Liu S, Wu LC, Pang J, Santhanam R, Schwind S (2010). Sp1/NFkappaB/HDAC/miR-29b regulatory network in KIT-driven myeloid leukemia.. Cancer Cell.

[34] Wu LC, Goettl VM, Madiai F, Hackshaw KV, Hussain SR (2006). Reciprocal regulation of nuclear factor kappa B and its inhibitor ZAS3 after peripheral nerve injury.. BMC Neurosci.

[35] Liu S, Liu Z, Xie Z, Pang J, Yu J (2008). Bortezomib induces DNA hypomethylation and silenced gene transcription by interfering with Sp1/NF-kappaB-dependent DNA methyltransferase activity in acute myeloid leukemia.. Blood.

[36] Hong JW, Allen CE, Wu LC (2003). Inhibition of NF-kappaB by ZAS3, a zinc-finger protein that also binds to the kappaB motif.. Proc Natl Acad Sci USA.

